# Radioecological characteristics of Siberian roe deer (*Capreolus pygargus* Pal., 1771) inhabiting locations of nuclear weapon tests

**DOI:** 10.1371/journal.pone.0308518

**Published:** 2024-09-17

**Authors:** Andrey Panitskiy, Asem Bazarbaeva, Symbat Baigazy, Ivan Alexandrovich, Natalya Larionova

**Affiliations:** NNC RK, Institute of Radiation Safety and Ecology, Kurchatov, Kazakhstan; University of South Carolina, UNITED STATES OF AMERICA

## Abstract

This paper reports the activity concentrations of ^137^Cs, ^90^Sr, ^239+240^Pu, ^241^Am, and ^3^Н in the form of tritiated water (НТО) and organically bound tritium (ОBТ) in the tissues and organs of roe deer (*Capreolus pygargus* Pal., 1771) that inhabit the ‘Degelen’ test location of the Semipalatinsk Test Site. Tissues and organs were sampled from six deer by killing. The activity concentrations of specific radionuclides in the samples were measured using γ-, α-, and β-spectrometry. The radionuclide activity concentrations in the tissues and organs showed considerable variation, for example, 0.6–170 Bq kg^-1^ for ^137^Cs and 0.3–2.8×10^3^ Bq kg^-1^ for ^90^Sr. The activity concentrations of radionuclides in animal muscular tissue did not exceed permissible values for the meat of wild animals. The tissues and organs in the roe deer were arranged as follows in descending order of their ability to accumulate ^137^Cs and ^90^Sr: for ^137^Cs, muscular tissue–kidneys–lungs–spleen–heart–liver–bone tissue; for ^90^Sr, bone tissue–liver–lungs–muscular tissue–spleen–heart–kidneys. The activity concentrations of ^241^Am and ^239+240^Pu did not exceed the minimum detectable activity. Therefore, no quantitative values could be determined for ^241^Am, and the ^239+240^Pu activity concentration could be derived for only one sample: 0.5±0.1 Bq kg^-1^ (liver). The distribution pattern of these radionuclides in the tissues and organs of the roe deer could not be determined because of insufficient data. The HTO volumetric activity in the tissues and organs of the examined animals ranged from 2.6×10^−2^ to 77 kBq l^-1^; activity concentration of OBT, 3.0×10^−2^ to 16 kBq kg^-1^; and OBT-to-HTO ratios, 2.0×10^−3^ to 5.3×10^2^. This ratio can serve as an indicator of how long the examined animals stay in radioactively contaminated ecosystems. Within the ‘Degelen’ site, the activity concentrations of ^90^Sr and tritium, in the form of HTO and OBT, are expected to be high in the bone tissues, soft tissues, and organs, respectively.

## Introduction

**Fig 1:**
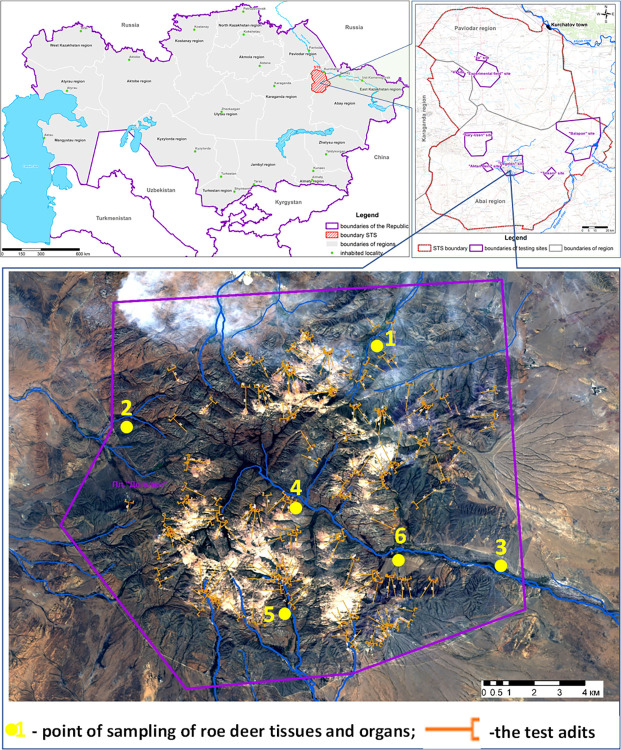
Location of the ‘Degelen’ site [3].

Nuclear weapons were tested at various test locations of the Semipalatinsk Test Site (STS) from 1949 to 1991 [1]. The ’Degelen’ site was one of them. It is located in the Degelen mountain range in the southern part of the test site (Fig 1) [2].

This mountain range is dome-shaped with an 18 km elevation and approximately 300 km^2^ diameter. The absolute elevations of the mountain range do not exceed 1000 m. At the ’Degelen’ site, underground nuclear tests were conducted in horizontal mine workings–adits. From 1961 through 1989, 209 nuclear tests were conducted in 181 test adits. Currently, all adits are sealed as part of activities on the elimination of nuclear infrastructure [4]. A radioecological survey of the ’Degelen’ test location showed that most of its territory is not contaminated with radionuclides. More than 90% of radioactivity produced during nuclear testing is concentrated in the cavities of test adits. However, elevated concentrations of radionuclides were detected in environmental compartments (soil, water, and vegetation) [5]. They were formed in off-normal situations during nuclear tests; they accumulated in the environment after the adits were opened after testing and carried away from the adit cavities by water.

Radioactive ground contamination associated with emergencies exhibits specific features such as the localization of man-made radionuclides in a relatively small area (0.01 km^2^ or smaller) and their concentration on the soil surface without any significant penetration deep down the soil horizon.

Radioactive contamination of near-mouth sites occurred as a result of adit unsealing and dumping of contaminated soil. Adits were opened to check the parameters of a nuclear test and for secondary use. Radioactive contamination in such adits is characterized by small areas. The height of radioactive dumps varies from 1 m to approximately 20 m. The most contaminated areas of the ‘Degelen’ site are near-mouth sites of adits with water streams. Long-term monitoring studies show that the radionuclides continue to be carried by water from cavities of nuclear blasts. In total, 8 to 12 such adits have been recorded at the ‘Degelen’ site during different years, depending on weather conditions. The maxima for the radioactivity concentrations in the water of streams from various adits were 8.2×10^2^ Bq l^-1^ for ^137^Cs, 2.1×10^3^ Bq l^-1^ for ^90^Sr, 6.4 Bq l^-1^ for ^239+240^Pu, 2.6 Bq l^-1^ for ^241^Am, and 9.9×10^5^ Bq l^-1^ for ^3^Н [5, 6]. Radioactive contamination typically reaches its maxima close to adit entries, near the outlet of water streams onto the daylight surface. As the distance from adit entries increases, ground contamination is reduced [2]. Thus, wild animals consuming water from radioactive creeks and foraging from radioactively contaminated ecosystems may be exposed to radioactive contamination. Our previous report described the activity concentrations of radionuclides in wild animals inhabiting the ‘Degelen’ test location [7, 8]. However, these animals, lizards and mouse-like rodents, were not game animals. No radioecological research has been undertaken to study species of wild game that inhabit test locations. The ’Degelen’ site is inhabited by various species of wild animals used in commercial and amateur hunting. One such species is the Siberian roe deer (*Capreolus pygargus* Pal., 1771). Its meat is consumed by the locals. The main goal of this paper was to perform a radioecological evaluation of the Siberian roe deer that inhabit the ‘Degelen’ test location and determine whether it is safe to consume in terms of radiation effects. Information on the distribution of radionuclides across tissues and organs in roe deer may be of interest to a wide range of researchers. Moreover, this species is listed as a reference animal as per RAPs ICPR 2008 [9].

## Materials and methods

### Field activities

At the ‘Degelen’ site, biological tissues were sampled from dead roe deer to determine the content of man-made ^137^Cs, ^90^Sr, ^239+240^Pu, ^241^Am, and ^3^Н in the form of tritiated water (НТО) and organically bound tritium (OBT).

Samples were collected from pre-killed roe deer. For this purpose, a permit was obtained to take away six roe deer for scientific purposes; this permit enables the use of animals for scientific, cultural and outreach, educational, and aesthetic purposes, as well as prevents epizooty (No.: KZ44VEP00133298; date of issue: 08/22/2022). Samples from the muscular and bone tissue and internal organs such as the heart, lungs, liver, and kidneys were collected from the roe deer. In certain cases, the horns were sampled. Altogether, 43 samples were collected, frozen, and sent to laboratories. These samples were selected because the muscular tissue and internal organs are consumed by humans and the bone tissue and horns can provide information on radionuclide distribution in animal tissues and organs. In addition, ^137^Cs is accumulated mainly in soft tissues (potassium counterpart) [10] and ^90^Sr in the bone tissue (calcium counterpart) [11].

### Laboratory research

#### Radionuclide analysis

^137^Cs and ^241^Am activity concentrations were measured on a γ-spectrometer, using a standard procedure [12]. The biological samples collected were pre-dried at 90°C or a lower temperature in ovens prior to analysis. Thereafter, they were charred at 400°C. The charred samples were ground and sieved through a 0.1 mm mesh to be thoroughly homogenized. A γ-spectrometer, Canberra GX-2020 (CANBERRA, USA), was used to measure ^137^Cs and ^241^Am activity concentrations in the charred samples, placed in a plastic container shaped like a 94 mm straight cylinder. The measurement time was longer than 60 min. The minimum detectable activity (MDA) for the ^137^Cs isotope was 0.3 Bq kg^-1^ and ^241^Am, 0.06 Bq kg^-1^. The γ-spectrometer was calibrated using calibration sources: IAEA-RGU-1 Uranium Ore diluted, IaEa-RGTh-1 Thorium Ore, and IAEA-RGK-1 Potassium Sulfate. The samples analysed using the γ-spectrometer were radiochemically prepared. The ^90^Sr activity was determined from the ^90^Y activity using the Quantulus 1220 ultra-low background liquid scintillation spectrometer-radiometer (using Cherenkov radiation). The chemical yields of Sr and Y carriers were determined using atomic emission spectrometry [13]. The MDA of the ^90^Sr isotope was 0.6 Bq kg^-1^. ICP-AS calibration was accomplished using multi-element solutions of reference standards (Inorganic Ventures IV-ICP-MS-71A). ^239+240^Pu was extracted and isolated through four main stages: complete chemical digestion, removal of interfering radionuclides, preparation of counting samples, and determination of radionuclide activity concentrations. A counting sample (α-source) was prepared by electrolytic precipitation of Pu onto a steel planchet for 60 min at 1.4 А and 12 V. ^239+240^Pu activity concentration was measured using an α-spectrometer with a solid-state detector, Alpha-Analyst (CANBERRA, USA) [14]. The minimum detectable activity for the ^239+240^Pu isotope was determined to be 0.01 Bq kg^-1^. The α-spectrometer was calibrated using a ^239^Pu calibration source manufactured by Source Inc. (Santa Fe, USA) and provided with a certificate of calibration (Ref. PO# 100060).

To determine the activity concentration of tritium in the form of HTO, free water was isolated from the muscular tissue and individual organs of animals in the form of condensate using a backflow condenser. The volume of free water isolated was at least 20 mL. Following the extraction of free water, the sample was dried to a constant mass, from which a 1–2 g aliquot was extracted and ashed using a ’Sample Oxidizer’ unit (PerkinElmer, USA). The activity concentration of OBT was measured in the water obtained after sample ashing. The activity concentration of tritium was measured using liquid scintillation spectrometry on a Quantulus 1220 spectrometer (Perkin Elmer, USA) [15]; a scintillation cocktail, Ultima Gold LLT, was added to natural samples at a 1:4 ratio (sample: scintillator) (registration efficiency for tritium, 0–18 keV in the order of 60%). The measurement time was at least 2 h. The measurements were processed using the Quanta Smart application. The minimum detectable activity of tritium was determined to be 0.7 Bq kg^-1^.

#### Mapping

Map documents were prepared using the software package ArcGIS on the basis of digitized maps of the Republic of Kazakhstan acquired from the Republican Public State Enterprise ‘National Mapping and Geodesic Fund’ of the Committee for Geodesy and Map-Making of the Ministry of Digital Development, Innovations and Aerospace Industry of the Republic of Kazakhstan (State Procurement Contract No. 02-19/122 dated 04/28/2020).

#### Quality control

Activity concentrations of radionuclides were measured using analytical and test equipment that was provided with calibration and test certificates (according to the Law of the Republic of Kazakhstan, dated 7 June 2000, No. 53-II ’On assurance of uniformity of measurements’).

Two methods were used to monitor the quality of measurements for radionuclide activity concentrations in the tissues and organ samples of the roe deer. The quality and reproducibility of analytical results were evaluated by placing one ’replicate’ sample in each batch of test samples. The ’replicate’ sample was randomly selected from the total number of samples in the batch. Hypothetical cross-contamination of samples was evaluated by adding ’reference’ samples to each batch. The ’reference’ sample was prepared from biological materials with known activity concentrations of test radionuclides at the ’background’ level.

### Processing of results

The present study evaluated activity concentration ranges for ^137^Cs, ^90^Sr, ^239+240^Pu, ^241^Am, and ^3^Н in the form of НТО and OBT in the tissues and organs of roe deer (*C*. *pygargus* Pal., 1771). To assess the pattern of radionuclide distribution in tissues and organs, the activity concentration of a radionuclide was converted to the concentration ratio, expressed as a percentage of the maximum activity concentration of the radionuclide in an organ or tissue of the animal under consideration. Thus, in the concentration ratio, the maximum activity concentration of the radionuclide in a tissue or organ of the animal was equal to 1.

## Results and discussion

The activity concentrations of ^137^Cs, ^241^Am, ^90^Sr, and ^239+240^Pu are listed in Table 1.

**Table 1:** Radionuclide content in tissues and organs of roe deer (*C*. *pygargus* Pal., 1771) from the ‘Degelen’ site.

Animal No.	Tissue type	Activity concentration of radionuclides, Bq kg^-1^			
		^137^Cs	^241^Am	^90^Sr	^239+240^Pu
1	Muscular tissue	0.6±0.1	<0.06	0.7±0.4	<5.4×10^−3^
	Bone tissue	<0.3	<0.4	39±11	<0.16
	Heart	<0.3	<0.06	<0.4	<9.8×10^−3^
	Kidneys	2.5±0.5	<0.06	2.7±0.4	<1.2×10^−2^
	Lungs	1.1±0.2	<0.04	0.6±0.14	<5.1×10^−3^
	Liver	20.5±4	<0.05	1.1±0.2	0.5±0.1
	Spleen	1.7±0.3	<0.06	1.2±0.2	<1.3×10^−2^
2	Muscular tissue	<0.3	<0.08	1.0±0.2	<8.7×10^−3^
	Bone tissue	0.2±0.03	<0.10	0.4±0.2	<9.5×10^−3^
	Heart	0.2±0.04	<0.05	0.3±0.06	<3.8×10^−3^
	Kidneys	2.1±0.4	<0.1	0.2±0.1	<1.9×10^−2^
	Lungs	56±11	<0.1	1.0±0.2	<1.4×10^−2^
	Liver	0.2±0.04	<0.06	6.5±2.7	<5.1×10^−2^
	Spleen	3±0.6	<0.1	0.3±0.2	<1.3×10^−2^
3	Muscular tissue	1±0.2	<0.09	0.3±0.08	<7.3×10^−3^
	Bone tissue	<0.3	<0.4	31±6	<0.18
	Heart	0.3±0.1	<0.05	0.2±0.07	<3.8×10^−3^
	Kidneys	<0.3	<0.07	0.5±0.2	<0.06
	Lungs	0.2±0.03	<0.05	0.3±0.08	<9.3×10^−3^
	Liver	0.2±0.03	<0.04	0.2±0.07	<1.1×10^−2^
	Spleen	0.3±0.1	<0.10	1.0±0.4	<8.4×10^−3^
4	Muscular tissue	170±34	<0.06	15.8±2.4	<0.01
	Bone tissue	5.7±1.1	<0.3	(2.8±0.4)×10^3^	<0.25
	Heart	77±16	<0.08	2.6±0.4	<0.25
	Kidneys	130±30	<0.09	4.5±0.7	<0.03
	Lungs	57±12	<0.03	14.5±2.2	<0.03
	Liver	120±20	<0.1	1.6±0.3	<0.02
	Horns	2.6±0.5	<0.3	820±120	<0.55
5	Muscular tissue	0.4±0.08	<0.02	1±0.2	<0.01
	Bone tissue	<0.3	<0.2	35±5	<0.13
	Heart	0.2±0.03	<0.02	3.8±0.6	<0.02
	Kidneys	<0.3	<0.1	1.7±0.9	<0.06
	Lungs	4.9±1.0	<0.02	1.4±0.2	<1.5
	Liver	<0.3	<0.05	19.6±2.9	<0.03
	Spleen	5.0±1.0	<0.02	2.3±0.6	<0.04
6	Muscular tissue	17±3	<0.2	1.0±0.2	<2.0×10^−2^
	Bone tissue	3.1±0.6	<0.2	(1.0±0.2)×10^3^	<4.0×10^−1^
	Heart	17±38	<0.3	0.5±0.08	<8.0×10^−3^
	Kidneys	13±3	<0.2	8.0±1.2	<3.0×10^−2^
	Lungs	1.1±0.2	<0.02	0.8±0.12	<1.0×10^−2^
	Liver	6.2±1.2	<0.05	11±1.6	<1.0×10^−2^
	Spleen	3.9±0.8	<0.1	1.7±0.3	<3.0×10^−2^
	Horns	<0.3	<0.2	820±120	<3.0×10^−1^
**Permissible value for the meat of wild animals**		300	Not standardized	100	Not standardized

The specific activity concentrations of ^137^Cs in the tissues and organs of the roe deer (*C*. *pygargus* Pal., 1771) were 0.6–170 Bq kg^-1^. The maximum value was recorded for the muscle tissue. It does not exceed the permissible value of ^137^Cs activity concentration for the meat of wild animals, which is 300 Bq kg^-1^ [16]. On the basis of the mean concentration ratios of ^137^Cs in tissues and organs of six roe deer, a descending series was generated to describe the ability of the tissues and organs to accumulate this radionuclide (Table 2).

**Table 2:** Concentration ratio of ^137^Cs in different tissues and organs of roe deer.

Tissues and organs	^137^Cs radionuclide concentration ratio (percentage of maximum specific activity in tissues and organs)				
	^a^N	^b^AM	^c^SD	Min	Max
Muscular tissue	5	8.2×10^−1^	4.1×10^−1^	8.2×10^−2^	1
Kidneys	6	5.2×10^−1^	4.2×10^−1^	3.8×10^−2^	7.6×10^−1^
Lungs	6	4.5×10^−1^	4.2×10^−1^	6.5×10^−2^	1
Spleen	5	3.8×10^−1^	3.6×10^−1^	5.4×10^−2^	1
Heart	3	3.5×10^−1^	3.6×10^−1^	3.4×10^−3^	1
Liver	5	2.7×10^−1^	2.7×10^−1^	3.4×10^−3^	7.1×10^−1^
Bone tissue	3	7.3×10^−2^	9.6×10^−2^	3.0×10^−3^	1.8×10^−1^

^a^n, number of samples

^b^AM, arithmetic mean

^c^SD, standard deviation

The tissues and organs of the roe deer can be arranged in descending order of their respective ability to accumulate ^137^Cs, as follows: muscular tissue–kidneys–lungs–spleen–heart–liver–bone tissue. This series depicts the general tendency of ^137^Cs distribution in tissues and organs in the roe deer. Nevertheless, the maxima of ^137^Cs activity concentrations were noted in the muscular tissue and in the lungs, heart, and spleen. Cs is a potassium counterpart. Potassium is the major cation in the intracellular medium. Of the total potassium in mammals, approximately 90% is in the cytoplasm. Therefore, the muscular tissue (soft tissues and organs) serves as the main potassium inventory in the body. The potassium concentration in the bone tissue is not high, attributing to low ^137^Cs activity concentrations in the bone tissue of roe deer [10, 17].

The ^90^Sr activity concentration in the tissues and organs of the examined roe deer varies within 0.3–2.8×10^3^ Bq kg^-1^. The maximum value was noted in the bone tissue. The maximum value in soft tissues and organs does not exceed 14 Bq kg^-1^ (lungs), nor is the permissible value of ^90^Sr activity concentration for the meat of wild animals, 100 Bq kg^-1^ [16]. The overwhelming majority of the maxima for the activity concentrations of radionuclides were observed for the bone tissue, which is demonstrated by creating a series of tissues and organs arranged in descending order of their ^90^Sr concentration ratios (Table 3).

**Table 3:** Distribution of ^90^Sr concentration ratio in the tissues and organs of roe deer.

Tissues and organs	^90^Sr radionuclide concentration ratio (percentage of maximum specific activity in tissues and organs)				
	^a^N	^b^AM	^c^SD	Min	Max
Bone tissue	6	8.4×10^−1^	3.8×10^−1^	6.2×10^−2^	1
Liver	6	2.7×10^−1^	4.2×10^−1^	5.7×10^−4^	1
Lungs	6	3.7×10^−2^	5.9×10^−2^	8.1×10^−4^	1.5×10^−1^
Muscular tissue	6	3.6×10^−2^	5.9×10^−2^	1.0×10^−3^	1.5×10^−1^
Spleen	5	3.5×10^−2^	2.3×10^−2^	1.7×10^−3^	6.6×10^−2^
Heart	5	3.3×10^−2^	4.7×10^−2^	5.2×10^−4^	1.1×10^−1^
Kidneys	6	2.9×10^−2^	2.6×10^−2^	1.6×10^−3^	6.9×10^−2^

^a^N–the number of samples; ^b^AM–arithmetic mean; ^c^SD–standard deviation

The tissues and organs of the roe deer can be arranged in descending order of their respective ability to accumulate ^90^Sr, as follows: bone tissue–liver–lungs–muscular tissue–spleen–heart–kidneys. Sr is a calcium counterpart. The bulk of calcium in adult animals (90–99%) is present in the bone tissue, incorporated in hydroxyapatite crystals [17, 18]. Therefore, the highest ^90^Sr activity concentrations are observed in the bone tissue. However, the concentration ratio of this isotope is elevated in the liver, the reason for which is unclear. One might assume that because the liver serves as a ‘filter’ in the body, it may retain a certain amount of ^90^Sr.

No quantitative values of ^241^Am could be determined. ^239+240^Pu activity concentration was derived for only one sample (liver, 0.5±0.1 Bq kg^-1^). The distribution pattern of these radionuclides in the tissues and organs could not be determined because of insufficient data. The missing numerical values for ^241^Am and ^239+240^Pu activity concentrations in the tissues and organs of the roe deer freely grazing around the ‘Degelen’ site is attributable to the low-migration characteristics of radionuclides belonging to the transuranic series in food chains both in the ‘soil-to-plant’ and ‘plant-to-animal’ chain [19]. Thus, the content of ^241^Am and ^239+240^Pu in the daily diet of the roe deer is not sufficient for quantifying the activity concentrations of these isotopes in their tissues and organs.

The activity concentrations of tritium in the form of НТО and OBT are listed in Table 4.

**Table 4:** Tritium activity concentrations in (*C*. *pygargus* Pal., 1771) tissues and organs of roe deer from the ‘Degelen’ site.

No.	Tissue / organ type	^3^H (НТО), kBq l^-1^	^3^H (OBT), kBq kg^-1^	OBT-to-HTO ratios
1	Muscular tissue	0.03±0.004	16±2	533.3
	Bone tissue	0.08±0.009	4±0.4	50.0
2	Muscular tissue	0.05±0.008	16±2	320.0
3	Muscular tissue	77±10	0.2±0.03	0.003
4	Muscular tissue	16±2	0.05±0.01	0.003
5	Muscular tissue	16±2	0.03±0.004	0.002
	Bone tissue	4±0.4	0.08±0.01	0.02
6	Muscular tissue	24±2	8.6±0.9	0.4
	Lungs	23±2	8.0±0.8	0.3
	Heart	21±2	8.1±0.8	0.4
	Liver	16±2	8.5±0.9	0.5

The HTO volumetric activities in the tissues and organs were 0.03–77 kBq l^-1^. The activity concentrations of tritium in the form of OBT ranged from 0.03 to 16 kBq kg^-1^. The maxima in both cases were observed in the muscular tissue. Tritium activity concentrations were higher in the soft tissues than in the bone tissue. The content of ^3^H in the meat of wild animals is not standardized [17]. If we compare the distribution of HTO and OBT between the muscular tissue and organs, the differences are obviously low and within the measurement error. In some tissues and organs, tritium in the form of HTO dominates by several orders of magnitude, while in others, OBT dominates by several orders of magnitude. Values of HTO and OBT activity concentrations within one order of magnitude were also noted. The calculated ratios of OBT-to-HTO activity concentration (OBT-to-HTO ratios) (Table 2) illustrate this difference and can serve as an indicator of how long the examined animals stayed in radioactively contaminated ecosystems [20, 21]. If the OBT-to-HTO ratios is orders of magnitude greater than 1, one can assume that the animal previously consumed a diet containing high activity concentrations of tritium for a long period and quite recently switched to a ’clean’ diet. In our study, higher values of OBT activity concentration were observed in tissues and organs because, relative to OBT, HTO has a shorter biological half-life, that is, the time required for a specific radionuclide accumulated in animal tissues or organs to decrease to half its initial concentration as a result of processes that exclude a physical decay [22]. If OBT-to-HTO ratios are orders of magnitude lower than 1, one can believe that a particular animal has only recently arrived at the tritium-contaminated ecosystem. Values of НТО and ОВТ concentrations within the same order of magnitude may indicate the duration over which the animal consumes a tritium-containing diet. The ratio in the present study is close to 1. The OBT-to-HTO ratios indicate that an equilibrium between OBT and HTO is absent in the roe deer, which suggests that animals do not feed on a radioactive diet on a permanent basis. Additionally, this observation is primarily related to the nonuniform distribution of radionuclides within the ‘Degelen’ test location. Generally, radionuclides are concentrated in ecosystem components containing radioactively contaminated water that flows from test tunnels; however, the radius of the daily activity of the roe deer expands beyond locally contaminated spots to various areas within the ‘Degelen’ site. Research into the nonhuman biota inhabiting Duke Swamp ecosystems showed that the OBT-to-HTO ratios for most wild animals were <1, which, in our opinion, suggests the absence of equilibrium conditions [23].

The activity concentrations of ^137^Cs, ^241^Am, ^90^Sr, and ^239+240^Pu experimentally derived in this paper are consistent with the estimated content of radionuclides in the meat of wild animals inhabiting STS, including areas outside the Degelen site, as previously reported by us [3], considering that animals inhabiting areas beyond test locations were evaluated. In either approach, no high values of radionuclide activity concentrations are expected in the meat of the roe deer, even though this research focused on roe deer inhabiting a location where the environmental components of the ecosystem are contaminated with radionuclides. Upon comparing the maxima derived for the ^137^Cs activity concentration in the muscular tissue of animals from the ‘Degelen’ site with the mean values of ^137^Cs activity concentration in the meat of roe deer and other hoofed animals derived during the period after the Chernobyl NPP accident, the latter proved to be orders of magnitude higher [24, 25]. For example, the mean values of ^137^Cs activity concentration in roe deer in the exclusion and evacuation zones were 1.7×10^4^ Bq kg^-1^ and 6.8×10^3^ Bq kg^-1^, respectively. The ^90^Sr activity concentration in the muscular tissue and internal organs of the roe deer in Chernobyl is comparable to the values derived for animals inhabiting the evacuation zone, ranging from 11 to 30 Bq kg^-1^ [26]. Among hoofed animals, in wild boar (*Sus scrofa* Lin., 1758) that inhabit areas exposed to radioactive contamination due to the Fukushima Daiichi Nuclear Power Plant (FNPP) incident (in the ex-evacuation zone), the mean ^137^Cs activity concentration was 470 Bq kg^-1^ [27]. Compared with these animals from the evacuation zones of the Chernobyl NPP and FNPP accidents, the roe deer inhabiting the Degelen test site exhibited lower activity concentrations of ^137^Cs, ^241^Am, ^90^Sr, and ^239+240^Pu in their soft tissues and organs. Within the ’Degelen’ test location, radionuclide contamination is associated with the washing away of man-made radionuclides from the cavities of test adits by water streams. Thus, within this site, high values of ^137^Cs, ^241^Am, ^90^Sr, and ^239+240^Pu activity concentrations in environmental components are only noted for ecosystems of radioactively contaminated water streams flowing out from adits and some creeks associated with these water streams, which is a small portion of the total test area [2]. When grazing freely, the daily diet of the roe deer is formed from food sources throughout the Degelen mountain range, including ‘dirty’ and ‘clean’ areas. The radionuclide activity concentrations in the soft tissues and organs, therefore, remain low owing to the amount and relatively short biological half-life of radionuclides consumed through dietary intake by the roe deer [22]. Compared with other tissues and organs, the bone tissue showed elevated values of ^90^Sr activity concentration, indicating that animals within the site consume a radioactively contaminated diet (plants, water, and soil). The excretion rate of ^90^Sr from the bone tissue is considerably lower than that from the soft tissues and organs. Therefore, we can expect elevated values for ^90^Sr activity concentration in the bone tissue long after a radioactively contaminated forage is excluded from the diet. Previously, authors showed that animals inhabiting STS, with small radii of daily activity (lizards and mouse-like rodents), exhibit relatively high values of ^137^Cs and ^90^Sr activity concentrations [7, 8].

The absence of quantitative values of ^241^Am and ^239+240^Pu activity concentrations in the tissue and organ samples of the roe deer may be due to the low values of feed transfer coefficients (F_f,_ d kg^-1^) for these radionuclides in the tissues and organs of hoofed (farm) animals [19]. F_f_ is computed as the mass or volumetric activity density in the receptor animal tissue or animal product (Bq kg^-1^ fresh weight) divided by the daily intake of a radionuclide (in Bq d^-1^). For example, according to the IAEA’s technical report TRS-472, F_f_ mean values of Pu isotopes for beef and mutton were 1.1×10^−6^ d kg-1 and 5.3×10^−5^ d kg^-1^, respectively. For Am, F_f_ single values did not exceed 5.0×10^−4^ d kg^-1^ [19].

The present research showed that roe deer within the ‘Degelen’ site are expected to show elevated values of ^90^Sr activity concentration in the bone tissue and fairly high values of tritium activity concentration both in the form of НТО and ОBT in the soft tissues and organs.

The data obtained on the levels of the major man-made long-lived ^137^Cs, ^241^Am, ^90^Sr, and ^239+240^Pu in tissues and organs of animals consumed by the public are important. These allow for the prevention of the existing speculations that these animals pose a hazard to man. This will foster the reduction of radiophobia among locals who are living in areas adjacent to STS. Thanks to findings, it is also possible to assess whether this species is acceptable to be chosen as a test objects in other research (for example, in radiobiological research). Subsequently, the information available on activity concentrations of radionuclides in question has to be supplemented with no threat to roe deer populations, for example, by collecting samples of biological tissues of roe deer from animals that died for various reasons (for example, following carnivores’ attacks or the demise during the mating season). Other species of hoofed animals inhabiting the territory of the ‘Degelen’ site also have to be addressed.

## Conclusions

The activity concentrations of ^137^Cs, ^90^Sr, ^239+240^Pu, ^241^Am, and ^3^Н in the form of НТО and ОBТ in the tissues and organs of the roe deer (*C*. *pygargus* Pal., 1771) were experimentally determined for STS on the basis of the corresponding values for the ‘Degelen’ test location. The activity concentration values of radionuclides in tissues and organs were 0.6–170 Bq kg^-1^ for ^137^Cs and 0.3–2.8×10^3^ Bq kg^-1^ for ^90^Sr; in the muscular tissue, these values did not exceed permissible values for the meat of wild animals. The activity concentrations of ^241^Am and ^239+240^Pu did not exceed the MDA. The only numerical value of ^239+240^Pu obtained was 0.5±0.1 Bq kg^-1^. Descending series were established to characterize the ability of tissues and organs to accumulate ^137^Cs and ^90^Sr in the roe deer inhabiting the ‘Degelen’ site. For ^137^Cs, this series is as follows: muscular tissue–kidneys–lungs–spleen–heart–liver–bone tissue. For ^90^Sr, this series is bone tissue–liver–lungs–muscular tissue–spleen–heart–kidneys. No values could be obtained for ^241^Am. The activity concentration for ^239+240^Pu was only derived for one sample: 0.5±0.1 Bq kg^-1^ (liver). The distribution pattern of these radionuclides in the tissues and organs of the roe deer could not be obtained because of insufficient data. The НТО volumetric activity in the tissues and organs of the examined animals varies from 2.6×10^−2^ to 77 kBq l^-1^. OBT activity concentration ranged from 3.0×10^−2^ to 16 kBq kg^-1^. OBT-to-HTO ratios were calculated to range from 2.0×10^−3^ to 5.3×10^2^. This ratio can serve as an indicator to determine how long the animals have stayed in radioactively contaminated ecosystems. Values available for OBT-to-HTO ratios demonstrate that these tritium forms are not in equilibrium, which indicates that tritium intake by animals is not constant.

Within the ‘Degelen’ site, elevated values of ^90^Sr activity concentration are expected in the bone tissue of the roe deer, and fairly high values of tritium activity concentration in the form of НТО and ОBT are expected in the soft tissues and organs.

It is important to conduct research on the activity concentrations of the major man-made long-lived radionuclides, ^137^Cs, ^241^Am, ^90^Sr, and ^239+240^Pu, in animal tissues and organs consumed by the public. These studies prevent the existing speculations that these animals pose a hazard to man and help reduce radiophobia among locals who live in areas adjacent to STS. The results from such studies enable researchers to assess the suitability of this species as a test object in other research (for example, in radiobiological research). Subsequently, information on radionuclide activity concentrations should be collected with no threat to roe deer populations, for example, by collecting samples of biological tissues from roe deer that died for various reasons (for example, carnivore attacks or demise during the mating season). Furthermore, other species of hoofed animals inhabiting the territory of the ‘Degelen’ site need to be studied.
